# Queen Specific Exocrine Glands in Legionary Ants and Their Possible Function in Sexual Selection

**DOI:** 10.1371/journal.pone.0151604

**Published:** 2016-03-17

**Authors:** Bert Hölldobler

**Affiliations:** 1 Social Insect Research Group, School of Life Sciences, Arizona State University, Tempe, Arizona, United States of America; 2 Biozentrum, Zoology II, University of Würzburg, Würzburg, Germany; Salford University, UNITED KINGDOM

## Abstract

The colonies of army ants and some other legionary ant species have single, permanently wingless queens with massive post petioles and large gasters. Such highly modified queens are called dichthadiigynes. This paper presents the unusually rich exocrine gland endowment of dichthadiigynes, which is not found in queens of other ant species. It has been suggested these kinds of glands produce secretions that attract and maintain worker retinues around queens, especially during migration. However, large worker retinues also occur in non-legionary species whose queens do not have such an exuberance of exocrine glands. We argue and present evidence in support of our previously proposed hypothesis that the enormous outfit of exocrine glands found in dichthadiigynes is due to sexual selection mediated by workers as the main selecting agents.

## Introduction

Army ants are usually defined by a specific syndrome of behavioral and reproductive traits which include obligate collective foraging, nomadism, and highly modified queens. The queens are called dichthadiigynes, they are permanently wingless and have a massive pedicel and large gaster which can become extremely physogastric [1–3]. The colonies propagate through fission of existing colonies and because the virgin queens are wingless, only the alate males depart on the wing from the home colonies and seek access to foreign colonies to mate with the wingless virgin queens. Although only very few instances of observed mating in army ants have been reported [2], genetic investigations revealed an extremely high mating frequency. For example in the neotropical species *Eciton burchelli* the mean observed and effective queen-mating frequency is 12.90 [4].

The so-called true army ants comprise the erstwhile subfamilies Aenictinae and Dorylinae in the Old World and Ecitoninae in the New World [2, 5, 6, 7], which are now subsumed under a more inclusive subfamily Dorylinae that contains army ants and their non-legionary relatives [8]. However, it has been noted that some species outside the notorious army ants convergently evolved the army ant syndrome. Among those legionary ants belong species of the ponerine genus *Onychomyrmex* and the leptaniline genus *Leptanilla*.

It is typical in such legionary or army ants for queens to be surrounded by a large retinue of workers. For *Eciton* queens 25 to 50 major workers and an additional large number of smaller workers have been reported to move close to the queen during colony emigrations. Similar observations were made with *Neivamyrmex* [9, 10] and *Aenictus* [11], and queens of *Leptanilla japonica* and *Onychomyrmex hedleyi* are also very attractive to workers, particularly when they are moving in emigration columns ([12], and personal observation by B. Hölldobler, respectively).

Presumably the queen’s attractiveness is due to chemical stimuli emanating from her body, and early histological studies by Whelden [13] and scanning microscopic surveys by Hölldobler and Rettenmeyer (reported in [14]) suggest an unusual endowment of *Eciton* queens with exocrine glands. However, queens of some other ant species which are not legionary ants but occasionally emigrate, are also very attractive to workers inside the nest and they are surrounded by a dense retinue of workers during occasional nest emigrations; striking examples are the weaver ants *Oecophylla longinoda* and *O*. *smaragdina* [15], or the leafcutter ants of the genus *Atta* [16]. Histological investigations of these queens did not reveal the enormous endowment of exocrine glands found in *Eciton* (Hölldobler unpublished). Therefore Franks and Hölldobler [14] hypothesized that in army ants the evolution of the queens’ and the males’ exocrine glandular systems may be driven by sexual selection. Because queens are flightless and never leave their colony, workers are in a position to choose which queen will take over each new colony which originates by colony fission, and the queens might be chosen by the workers based on her attractiveness indicating potential productivity. Gottwald [2] questions the validity of this hypothesis and argues these glands may just produce a queen signal that attracts the retinue of workers.

In the current paper we study or reexamine the exocrine glandular system in *Eciton*, *Neivamyrmex* and *Aenictus* army ants and we compare these with the glandular equipment of queens of some other legionary ant species, such as the ponerine species *Onychomyrmex* and the leptallinine genus *Leptanilla*. In all cases we compare the queens’ exocrine glands with those of the workers. In light of these results we will reconsider the hypothesis proposed by Franks and Hölldobler [14].

## Material and Methods

Queens of the following species were investigated: *Aenictus* sp., collected by Mark Moffett, Mt. Apo, Mindanao, Philippines, fixed Carnoy’s fixative; *Eciton hamatum*, collected by Carl W. Rettenmeyer in Limoncocha, Ecuador, and Robert Silberglied, Barro Colorado, Panama, fixed in Bouin; *Eciton rapax*, collected by Carl w. Rettenmeyer in Limoncocha, Ecuador, fixed in Bouin; *Neivamyrmex nigrescens*, collected by Stefan Cover and Bert Hölldobler in Florida, USA, fixed in Bouin, *Neivamyrmex carolinensis* collected by Christina Kwapich in Florida, USA, fixed in 75% ethanol; *Leptanilla japonica*, collected by Keichi Masuko, near Tokyo, Japan, fixed in Kahle’s fixative; *Onychomyrmex hedleyi*, collected by Robert Taylor and Bert Hölldobler near Lake Barrine, Queensland, Australia, fixed in Carnoy’s fixative.

For histological investigation we used different techniques, because this comparative study began more than 20 years ago and continued, with interruptions until recently. Specimens were embedded in methyl methacrylate and sectioned 6μm to 8μm thick with a D-profile steel knife on a Jung Tetrander microtome [17] and the sections were stained with Heidenhain Azan. Smaller objects were embedded in a water soluble plastic (JB-4 embedding kit, Polysciences, Inc., Pennsylvania) and sectioned 4μm thick with glass knives on a rotary microtome. Sections were stained with hematoxylin-eosin.

## Results

### Exocrine Glands in Queens of “True” Army Ants

#### Exocrine glands in *Eciton* queens: Cuticle glands

**Fig 1:**
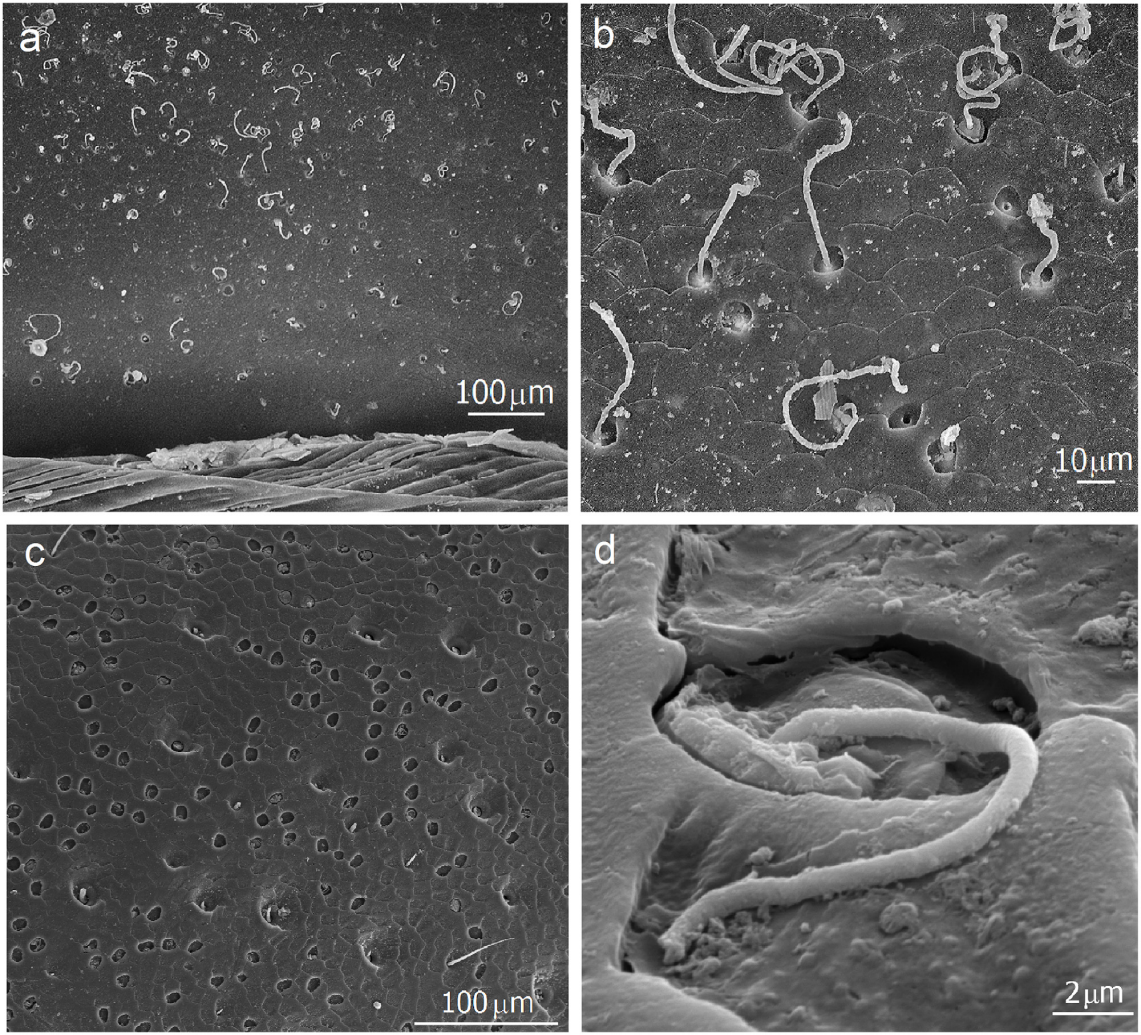
SEM micrographs of the openings of cuticle glands in Eciton *hamatum* queens. **a** and **b**: Abdominal tergite showing the dense assembly of gland pores with many showing secretions oozing out (modified from [12]). **c**: Cuticle glandular pores on the coxa. **d**: Secretions oozing out of a glandular pore on the coxa. The surface is covered with secretion.

**Fig 2:**
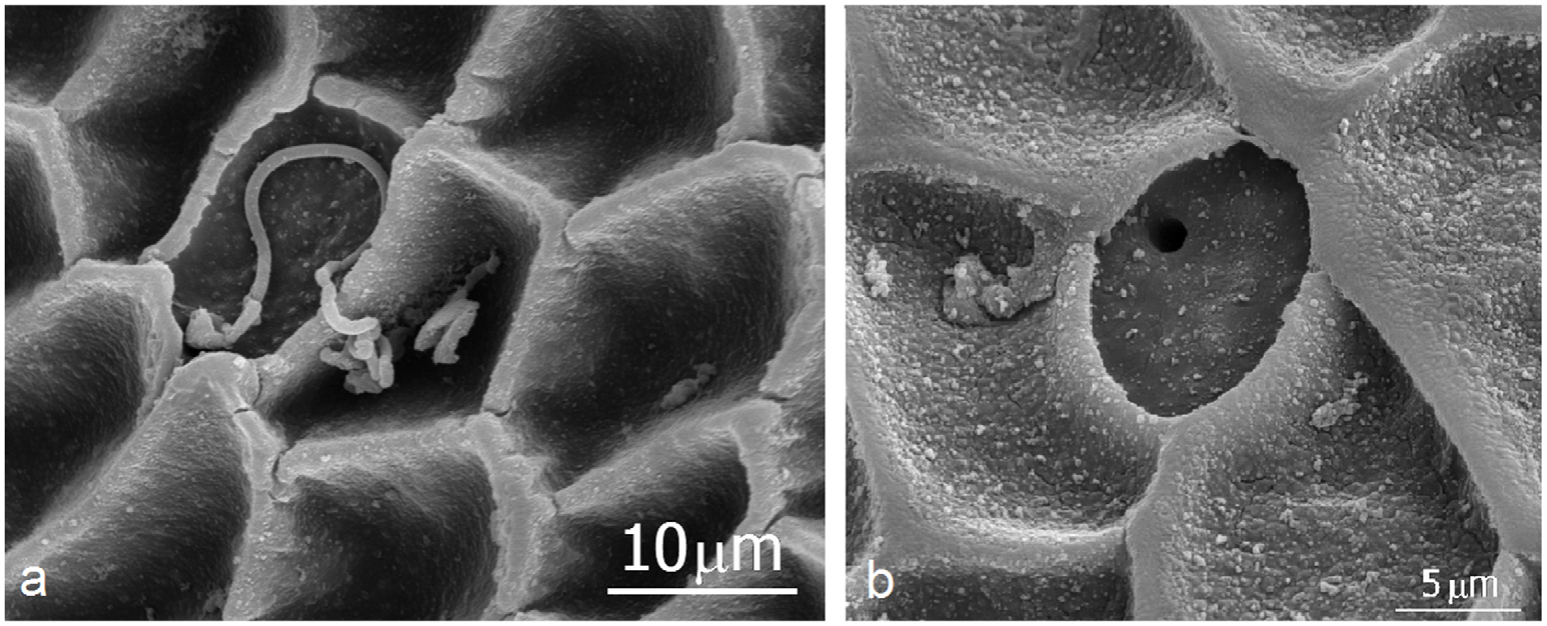
SEM micrographs of the post petiole cuticle of *Eciton rapax*. **a**: The glandular secretions within the cuticle cup structure. **b**: close up of the cuticle pore.

Our scanning electron microscopic studies of the gasters of *E*. *hamatum* and *E*. *rapax* queens confirm previous findings in *E*. *hamatum* of densely spaced glandular pores (distances 15μm to 50μm) on the entire cuticle surface. Coagulated secretions can be seen oozing out of the pores ([14] and this study) (Fig 1). The cuticle surface often appears to be patterned by cup structures which are in part covered with secretions. Removing the coagulated secretion exposes the glandular openings inside the cups (Fig 2). But not only the gaster is endowed with such dense assemblies of glandular pores, we found similar structures all over the body of *Eciton* queens, on thorax, legs, head, and mandibles. The following description of our findings is based on both species which have largely an identical glandular morphology.

**Fig 3:**
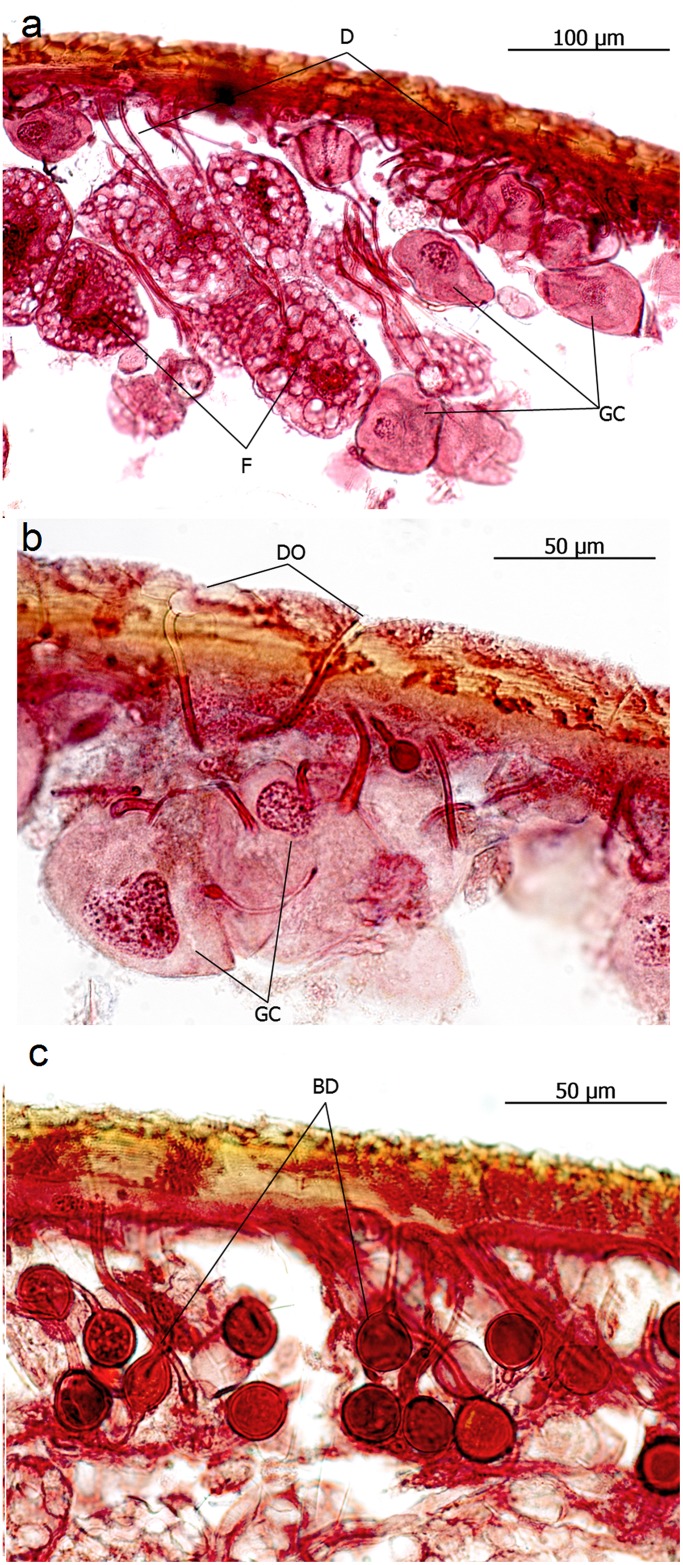
Longitudinal sections through the gaster of *Eciton hamatum* queens. **a**: Many glandular ducts (D) draining secretions from the glandular cells (GC) which are embedded in a dense assembly of fat cells (F), and open through the cuticle (DO in **b**). **c**: Often the ducts are inflated to form balloon shaped dilations (BD).

The histological investigations revealed dense layers of glandular duct cells opening through the cuticle. Many of the ducts exhibit balloon-like inflations near the cuticle openings (Fig 3). These duct cells drain the secretion of many glandular cells embedded in a rich fat body. Whelden [13] in his extensive histological study of exocrine glands in *Eciton* misinterpreted these inflated duct cells as “aeration tubes and chambers”.

**Fig 4:**
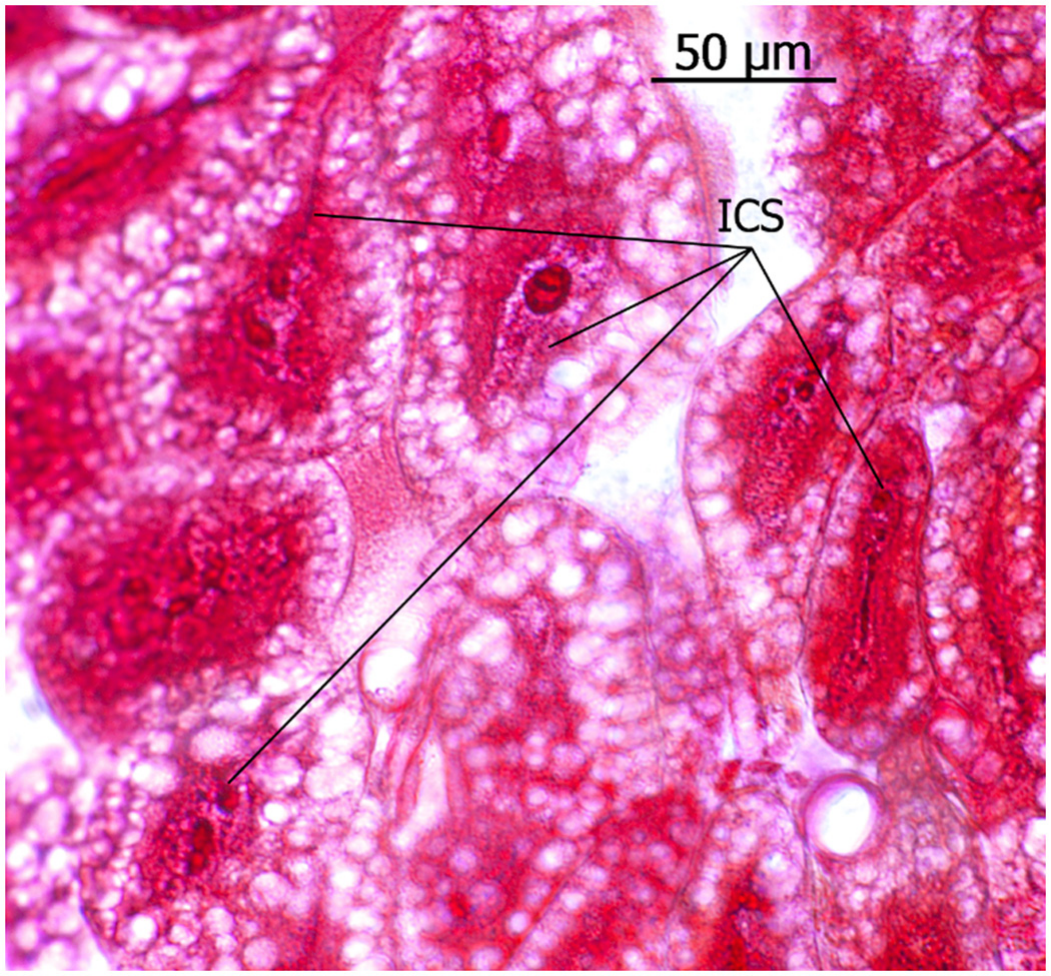
Cells within the fat body of *Eciton* queens. These cells feature an internal cell structure (ICS) resembling an end apparatus of duct cells.

A large number of the cells, which look like fat body cells because they are filled with many vacuoles, have distinct nuclei and an internal cell structure that resembles the end apparatus of an associated duct cell, but we were unable to unequivocally detect duct cell connections and we therefore assume these cells are also part of the very rich fat body (Fig 4).

#### Exocrine glands in *Eciton* queens: Intersegmental glands

**Fig 5:**
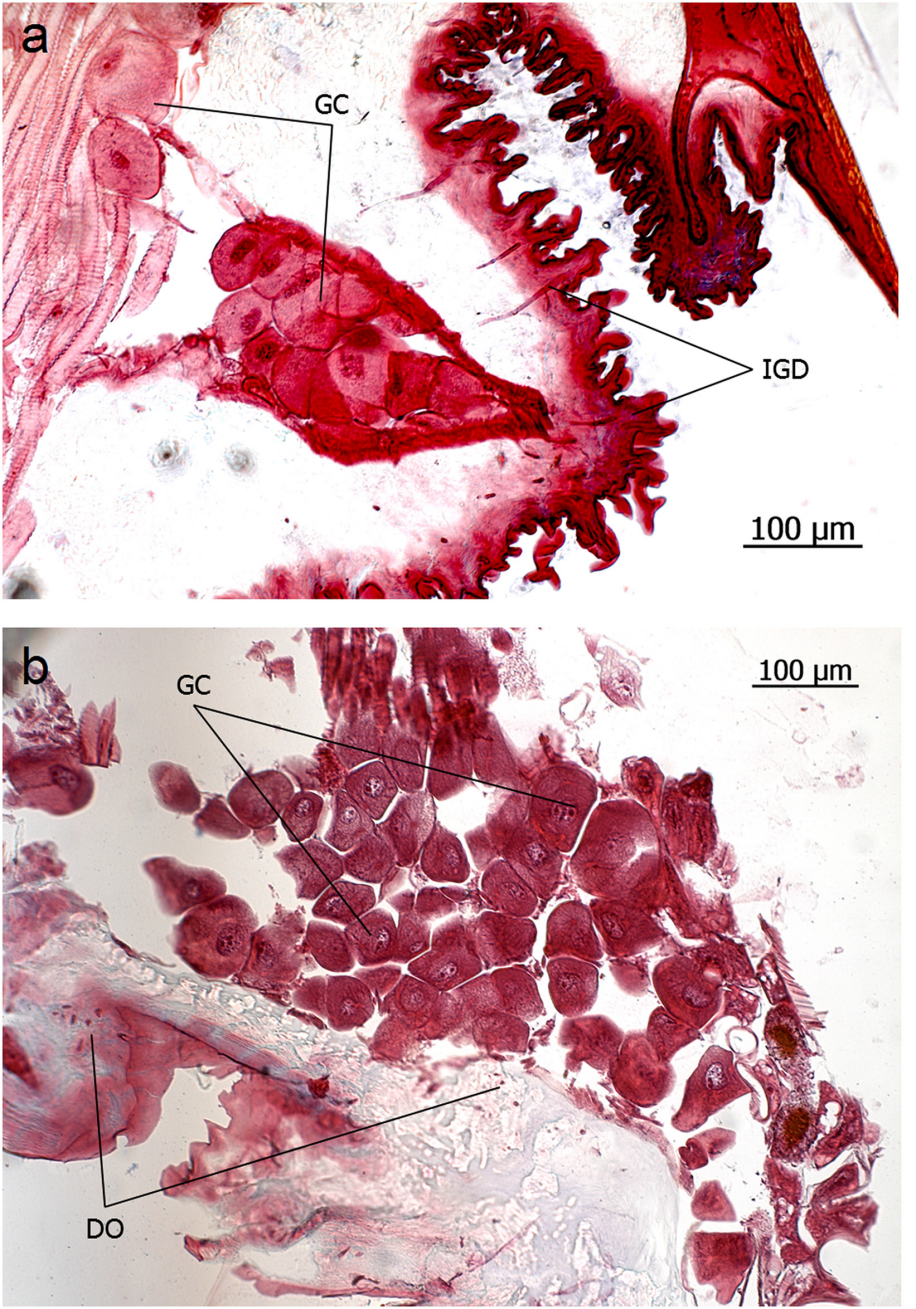
Intersegmental glands in *Eciton* queens. **a**: Intersegmental sternal gland cells (GC) of *E*. *hamatum*, opening through duct cells that penetrate the intersegmental membrane (IGD). **b**: Intersegmental tergal gland cells (GC) of *E*. *rapax* opening through duct cells that penetrate the intersegmental membrane (duct opening DO).

**Fig 6:**
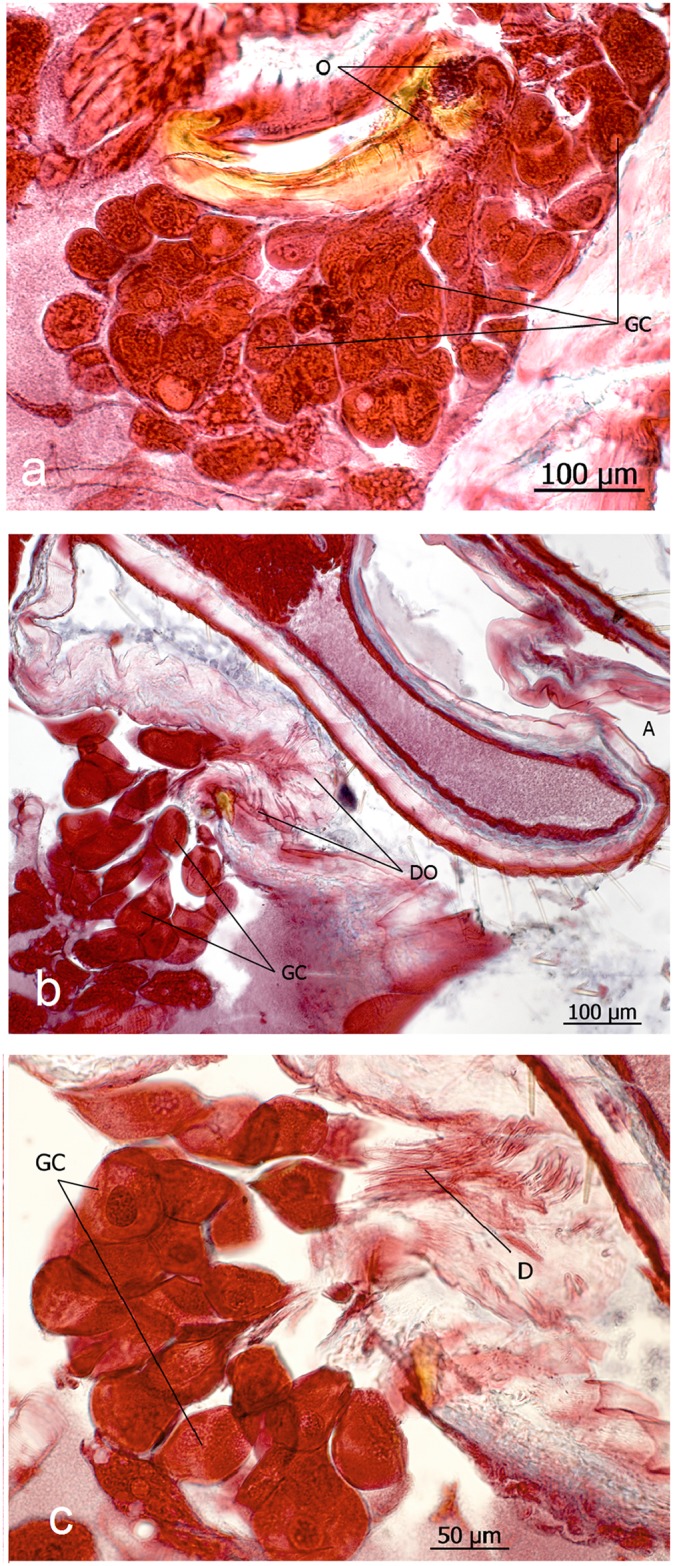
Abdominal exocrine glands in *Eciton rapax* queens. **a**: Oblong plate gland, the glandular cells (GC) of which are drained by duct cells that open (O) through the cuticle piece of the IXth sternite. **b** and **c**: Sternal gland duct cells (D) which open (DO) through the membrane located ventrally from the anus (A).

**Fig 7:**
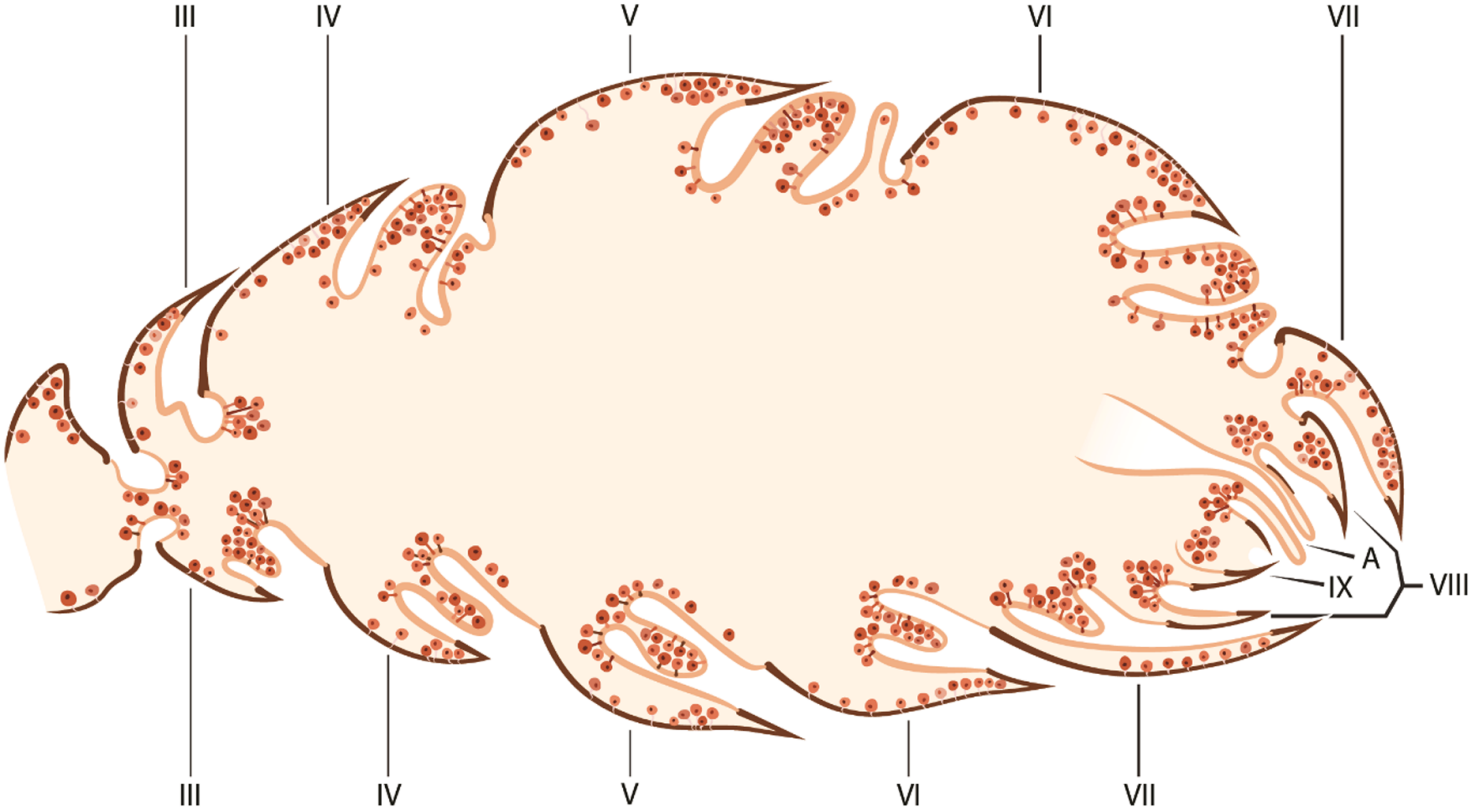
Schematic overview of the cuticle and intersegmental gland in an *Eciton* queen. Gland cells symbolized by dots. Roman numerals indicate abdominal segments. Anus (A).

Whelden [13] has reported intersegmental exocrine glands in all gaster segments of *Eciton* queens which appear to be particularly well developed in the first two gaster segments. We also found in each gaster segment dorso-lateral and ventro-lateral intersegmental glands, consisting of clusters of glandular cells that drain their secretions through associated duct cells that penetrate the thick intersegmental membrane (Fig 5). In addition we found such paired clusters of glandular cells in the segments which comprise the sting apparatus. We identified a spiracular plate gland (segment VIII), a quadrate plate gland (segment IX), a ventrally located oblong plate gland (segment IX ventral), the duct cells of which open, in contrast to the duct cells of the other plate glands, not through an intersegmental membrane, but, at least in part, through the cuticle (Fig 6a). There is possibly also a triangular plate gland, although we had difficulties unambiguously identifying the exact position of these paired glandular clusters. Finally we found a large paired sternal gland the duct cells of which penetrate the intersegmental membrane close to a small cuticle section most likely part of X^th^ sternite located ventrally from the anus (Fig 6b and 6c). The schematic illustration in Fig 7 reflects the amazing abundance of exocrine glands in the gaster of *Eciton* queens.

**Fig 8:**
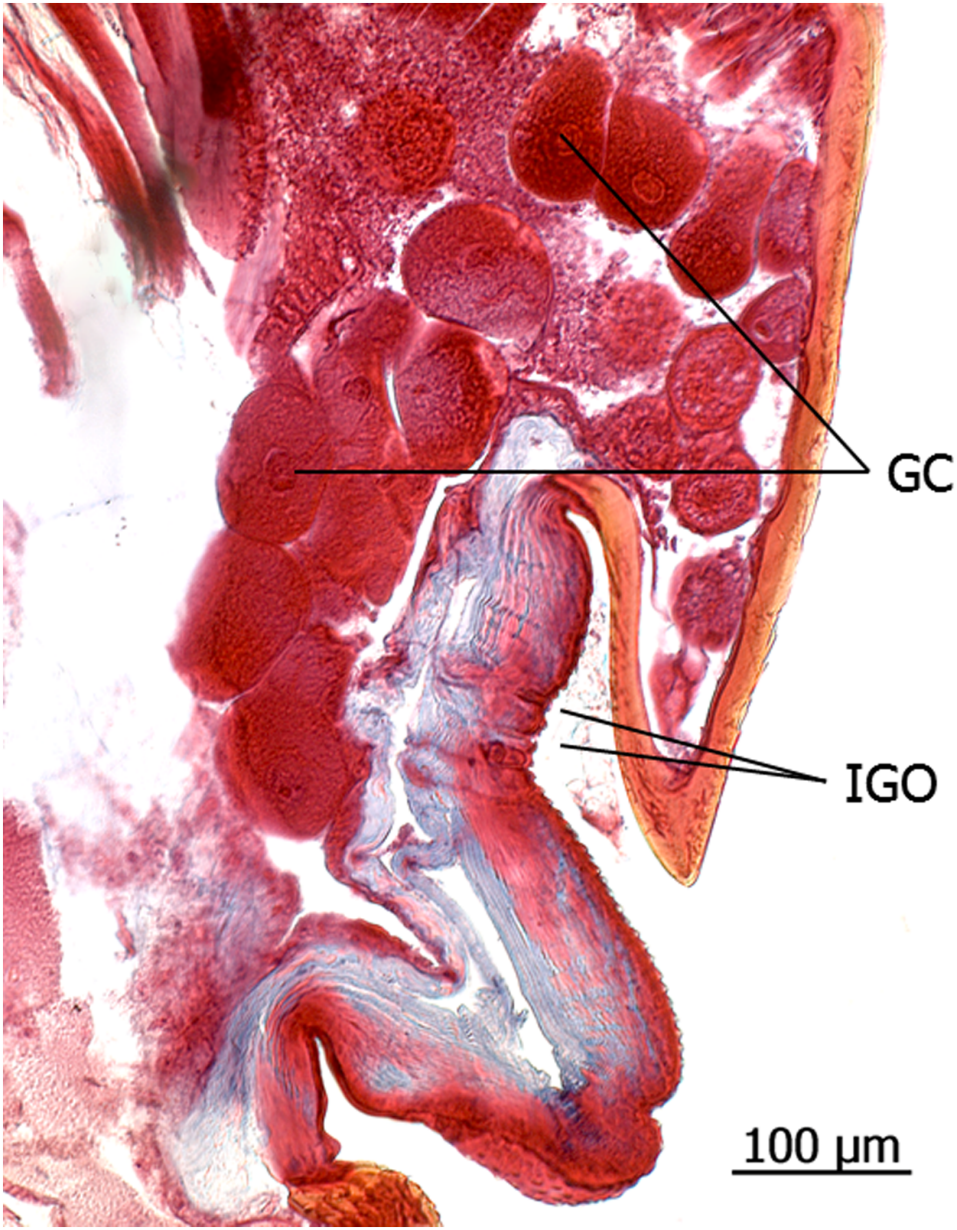
Intersegmental gland between thorax and coxa of an *Eciton hamatum* queen. The secretions of the glandular cells (GC) are drained by duct cells that penetrate and open through the intersegmental membrane (IGO).

**Fig 9:**
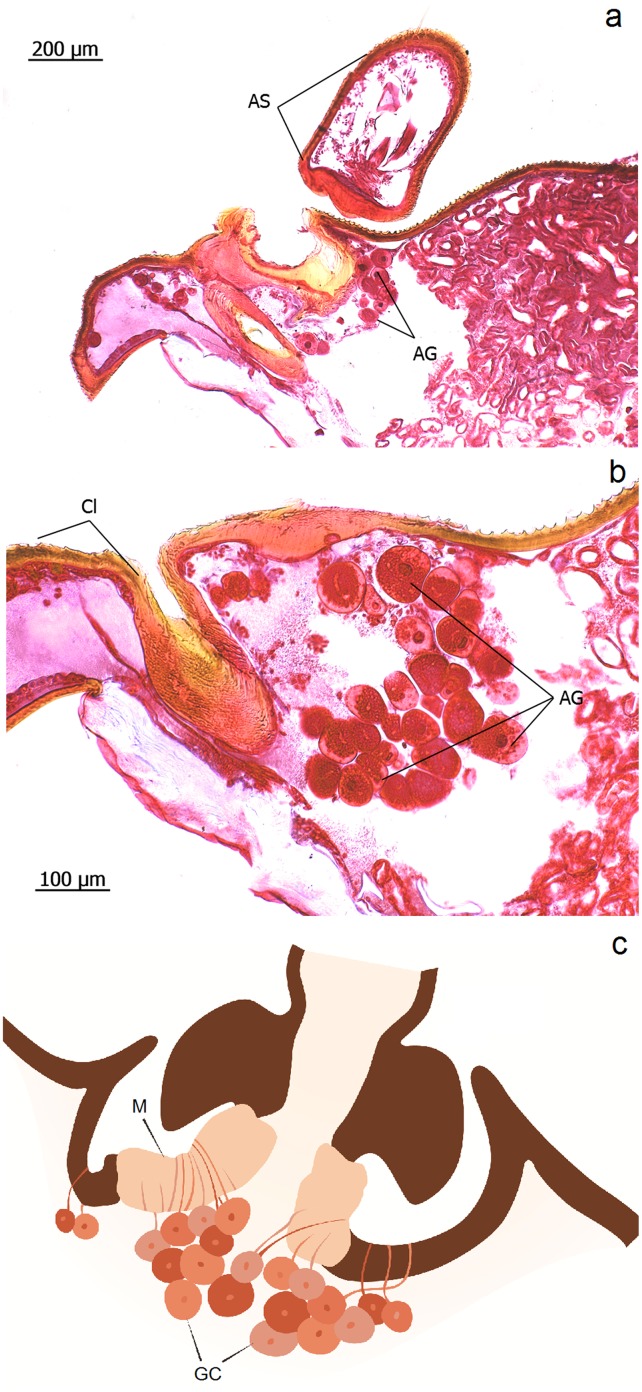
Intersegmental antenna gland in *Eciton rapax* queens. **a**: Antenna scape (AS), intersegmental antenna gland (AG). **b**: Close-up of intersegmental antenna gland; clypeus (Cl). **c**: Schematic drawing of the intersegmental antenna gland; membrane (M) connecting antenna (A) with head case, glandular cells (GC).

**Fig 10:**
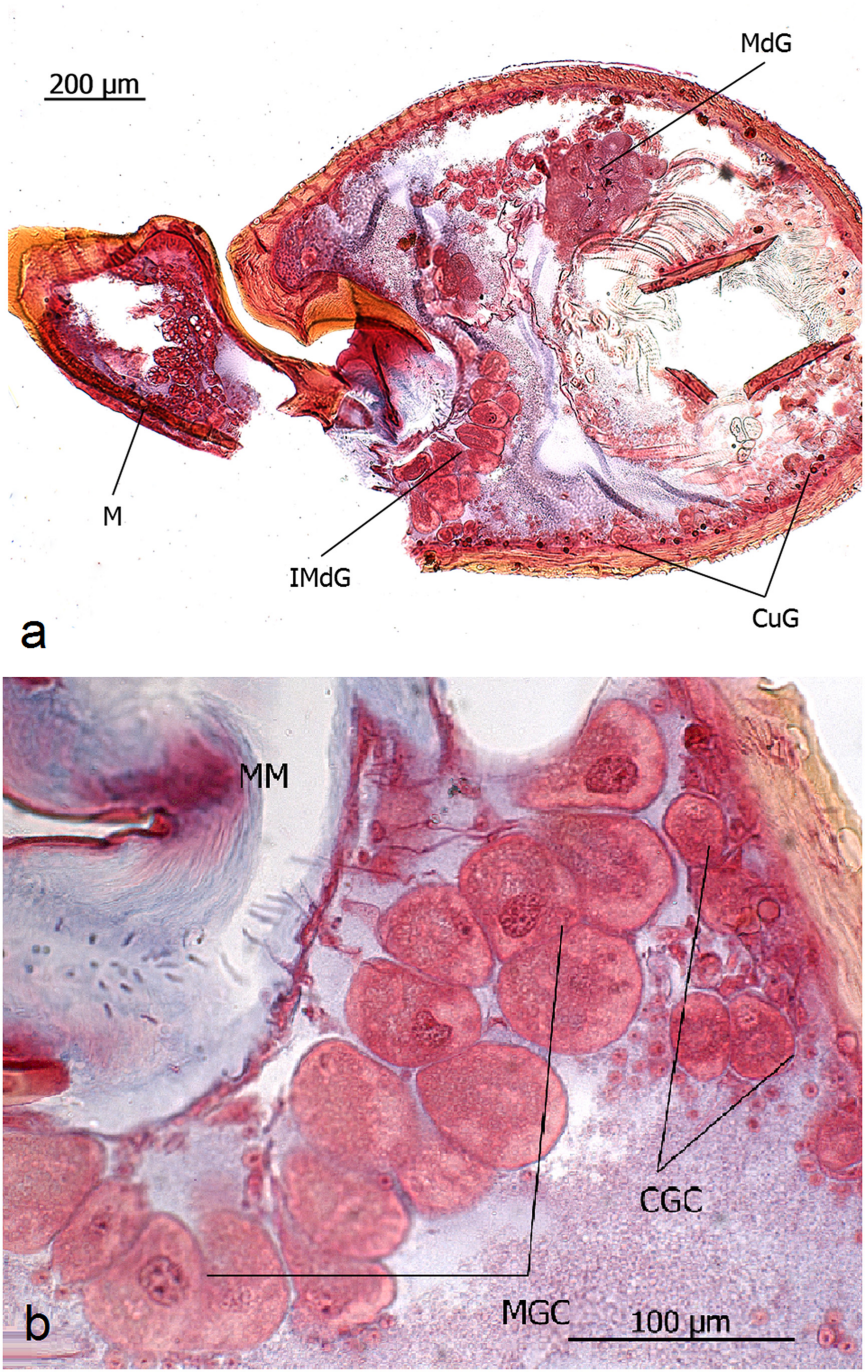
Intersegmental mandible gland in *Eciton* queens. **a**: Mandible (M), mandibular gland (MdG), cuticular glands (CuG), intersegmental mandible gland (IMdG). **b:** Close-up view of intersegmental mandible gland. Membrane connecting head case and mandible (MM), cuticular gland cells (CGC), mandibular gland cells (MGC).

However, it is not only the gaster of *Eciton* queens which is richly endowed with exocrine glands. Whelden [13] already reported intersegmental glands in the thorax between gaster and post-petiole, post-petiole and petiole, petiole and thorax, and also between thorax and coxa of all three pairs of legs. Our studies confirm this (Fig 8); in addition we found intersegmental glands literally in all segments connected by a membrane, such as between thorax and head, head-case and antenna (Fig 9), and head-case and mandible (Fig 10). The latter could erroneously be identified as mandibular gland; however, as will be shown below, this intersegmental mandible gland is not identical to the mandibular gland proper.

#### Exocrine glands in *Eciton* queens: Head

**Fig 11:**
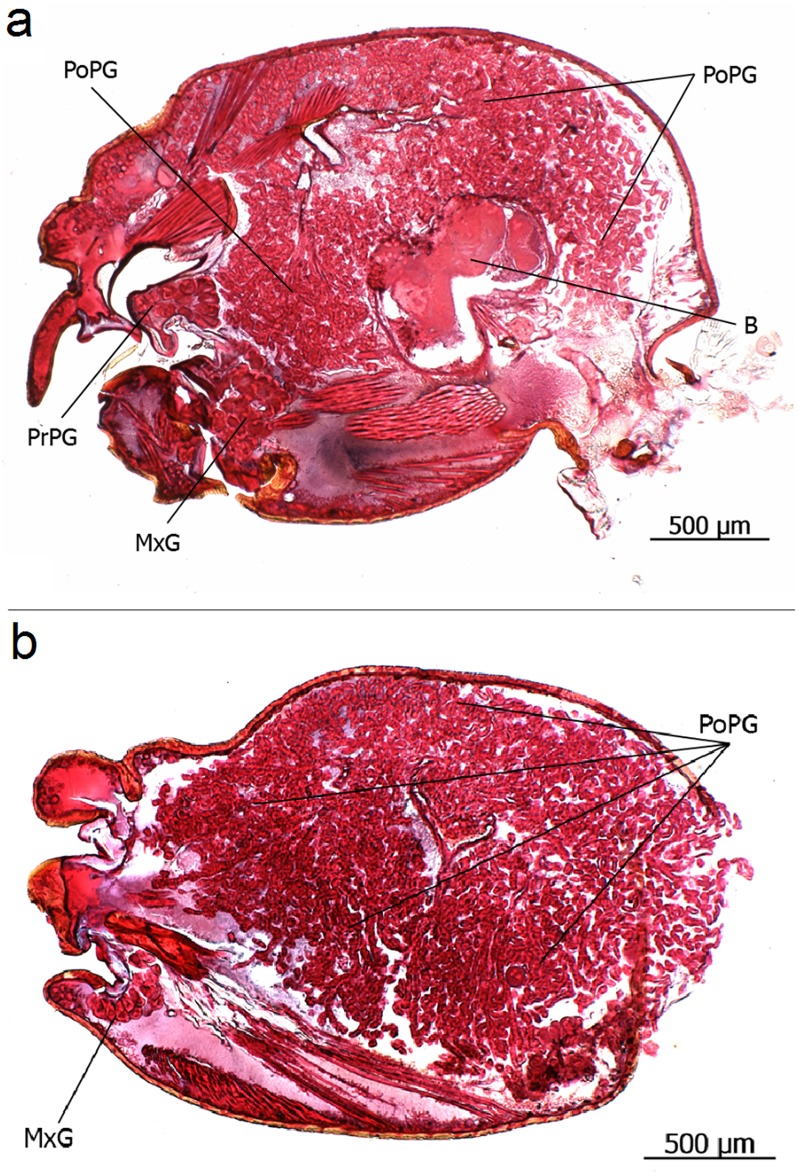
Longitudinal section through the head of *Eciton hamatum* queens. **a**: Postpharyngeal gland (PoPG), brain (B), maxillary gland (MxG), propharyngeal gland (PrPG). **b**: The postpharyngeal gland (PoPG) occupies large portions of the head case.

**Fig 12:**
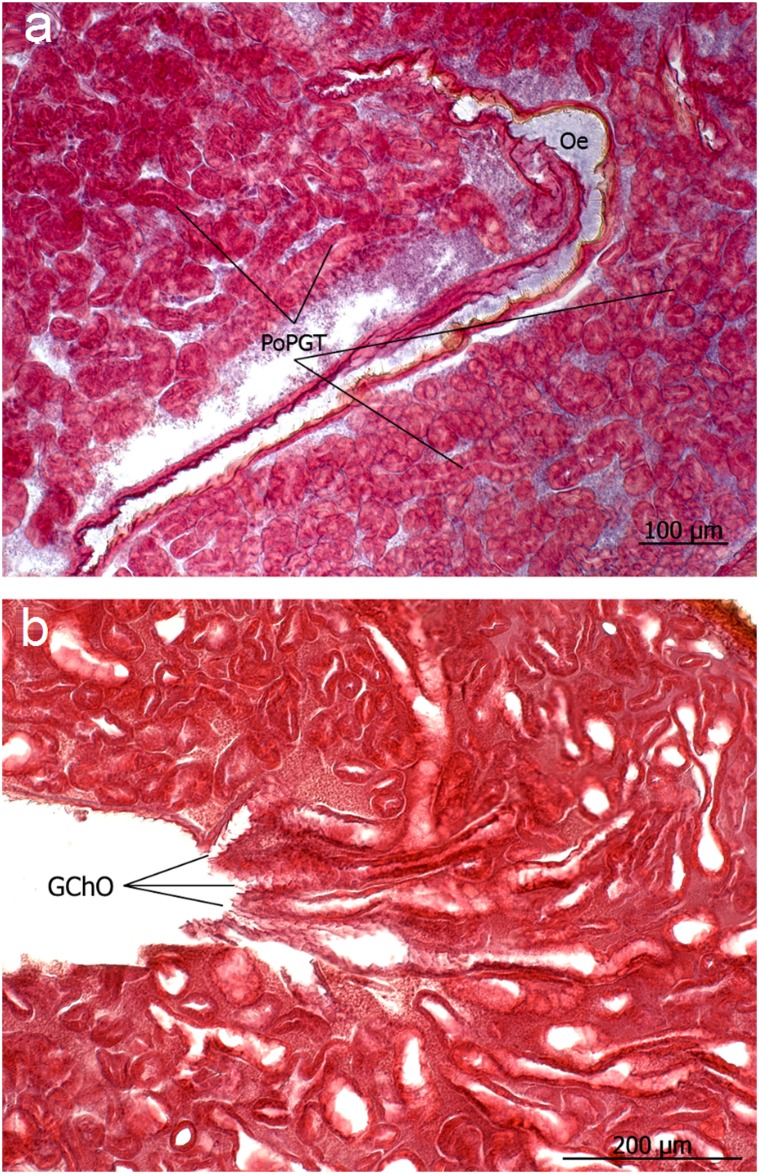
**a** and **b**: The numerous glandular tubes of the postpharyngeal gland (PoPGT) lined by an active glandular epithelium merge into glandular channels that open (GChO) into the oesophagus (Oe).

**Fig 13:**
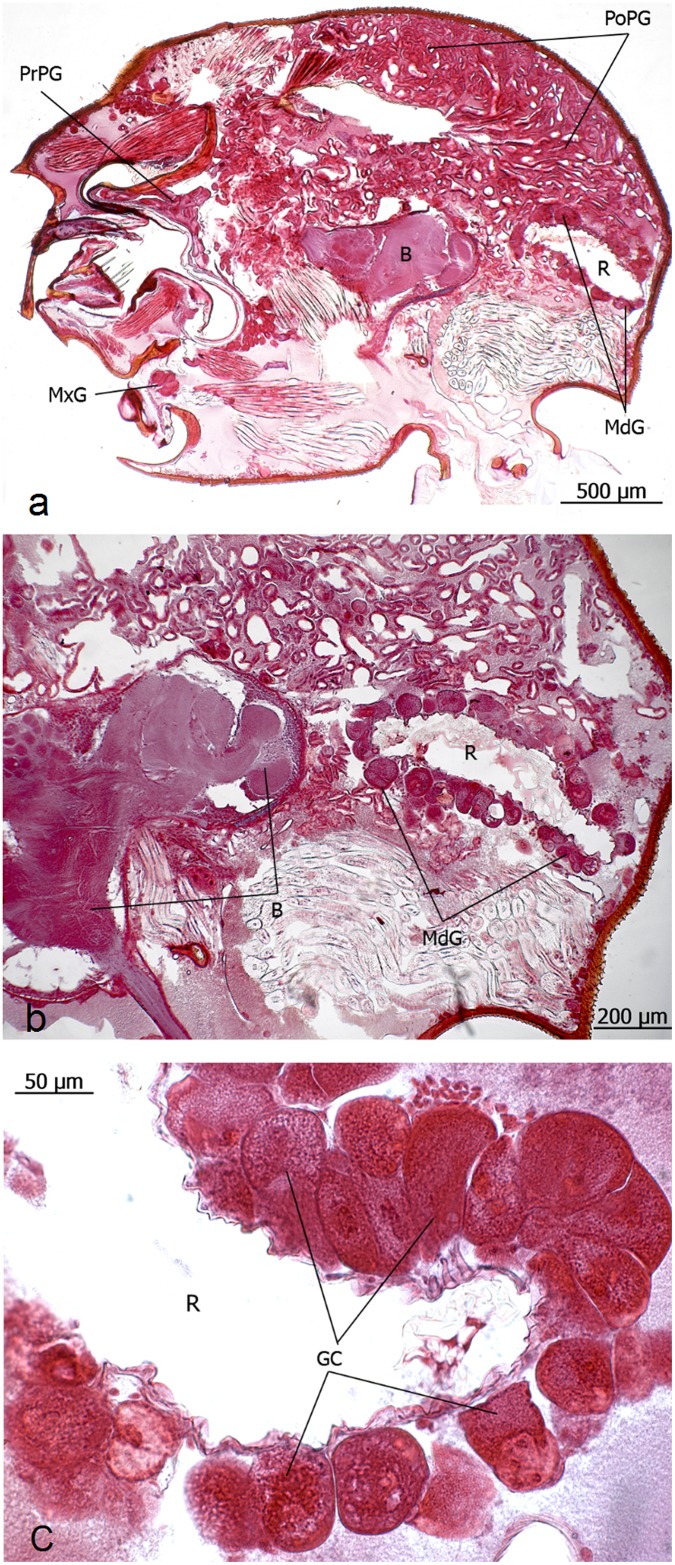
Longitudinal section through the head of an *Eciton rapax* queen. **a** and **b**: Propharyngeal gland (PrPG), postpharyngeal gland (PoPG), reservoir of mandibular gland (R), mandibular gland (MdG), maxillary gland (MxG), brain (B). **c**: close-up of the mandibular gland cells (GC) and reservoir (R).

**Fig 14:**
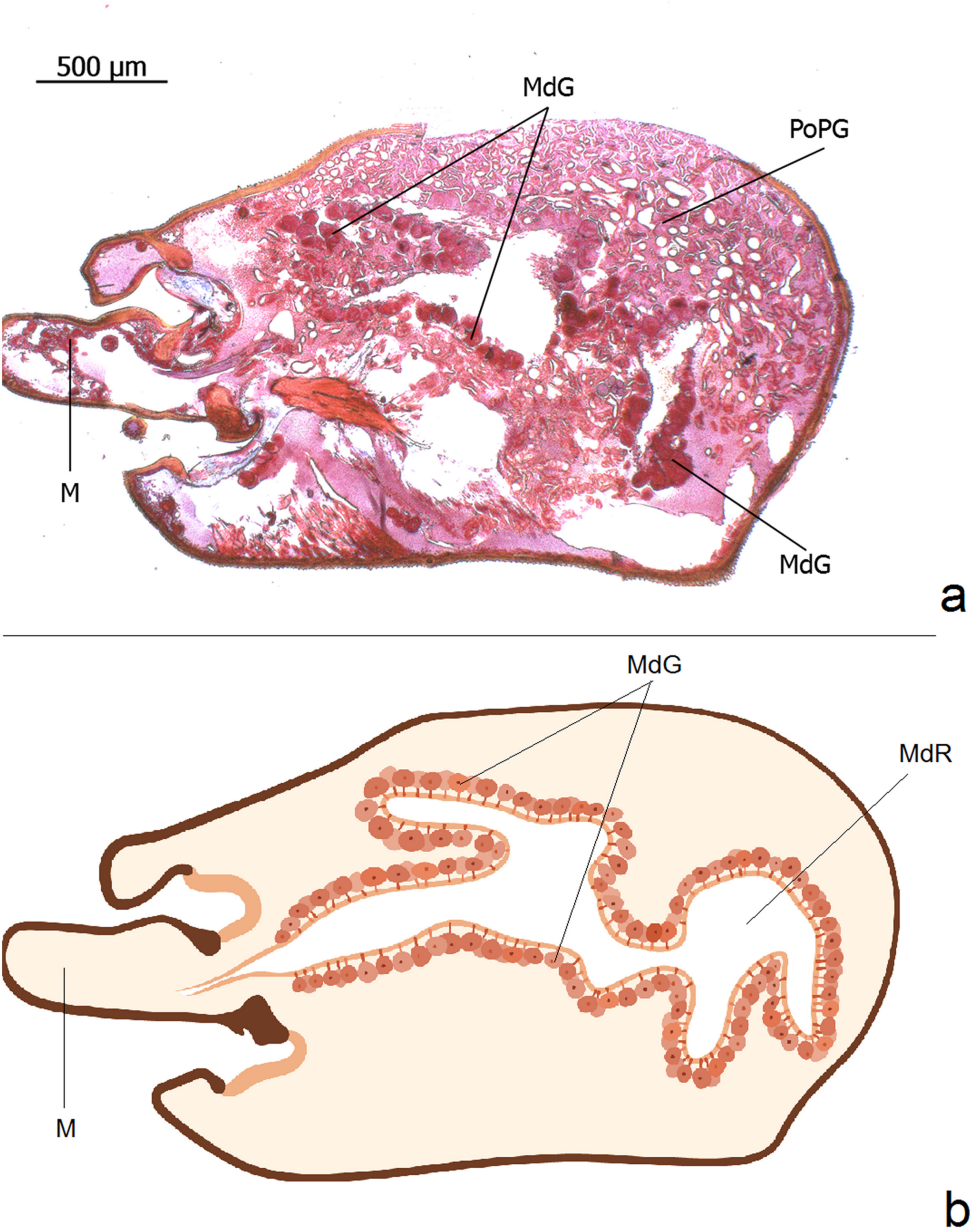
Longitudinal section through the head of an *Eciton rapax* queen showing larger portions of the mandibular gland. **a**: Mandibular gland (MdG), postpharyngeal gland (PoPG), mandible (M). **b**: Schematic illustration of the large mandibular gland reconstructed out of several serial longitudinal sections.

**Fig 15:**
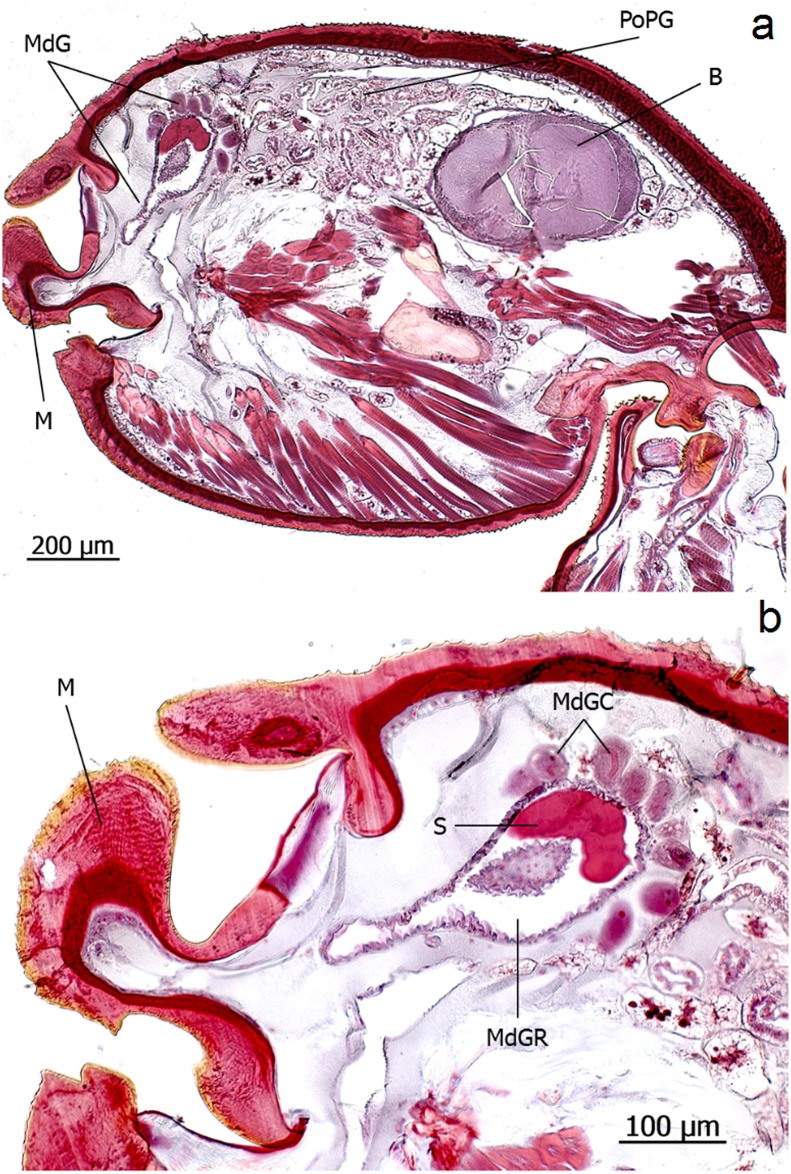
Longitudinal section through the head of an *Eciton hamatum* worker. **a**: Mandibular gland (MdG), postpharyngeal gland (PoPG), brain (B), mandible (M). **b**: mandibular gland cells (MdGC), mandibular gland reservoir (MdGR), mandibular gland secretion (S).

**Fig 16:**
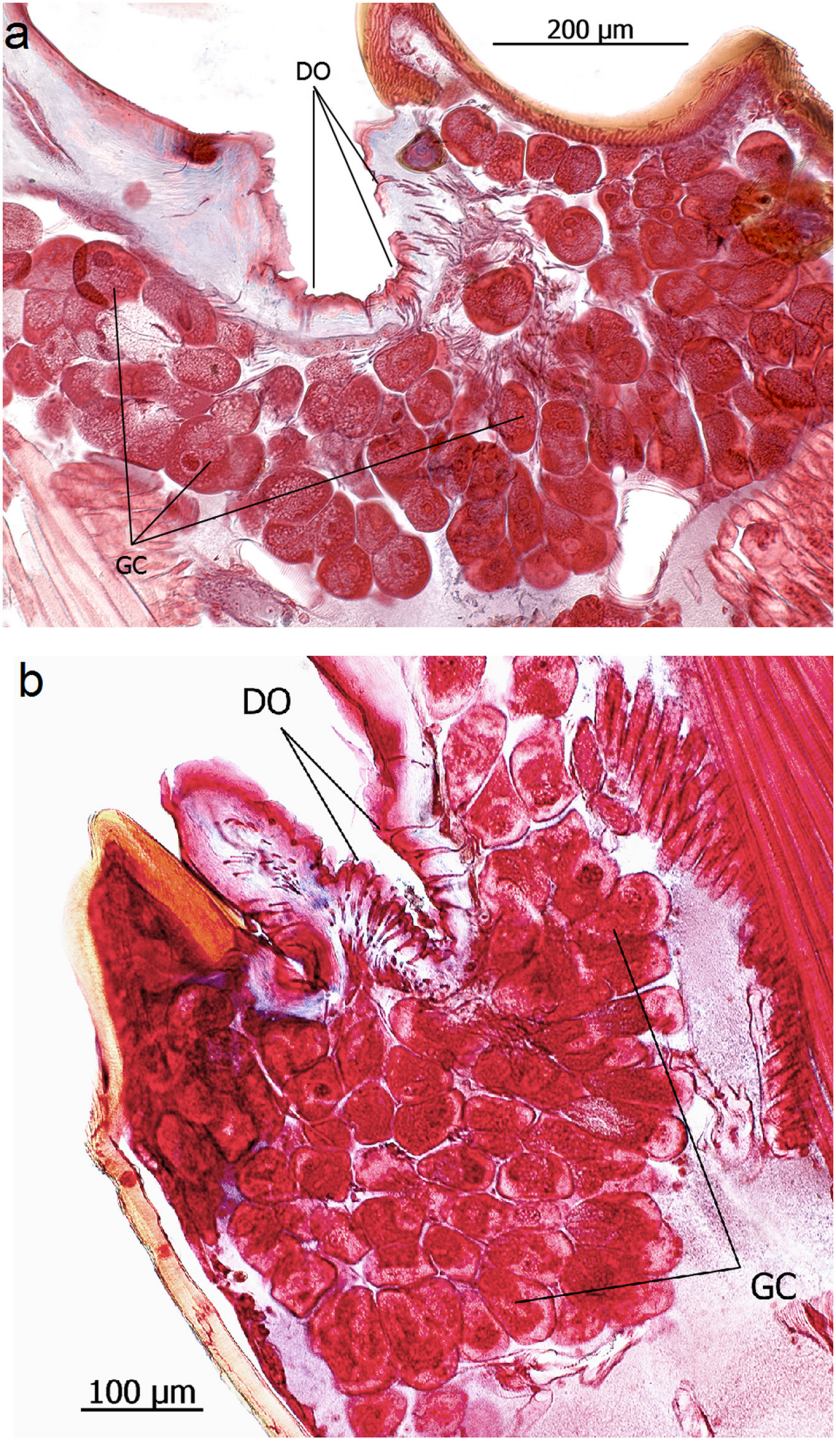
Maxillary glands of *Eciton* queens. **a**: *E*. *rapax*; **b**: *E*. *hamatum*. Glandular cells (GC), duct cell openings (DO).

**Fig 17:**
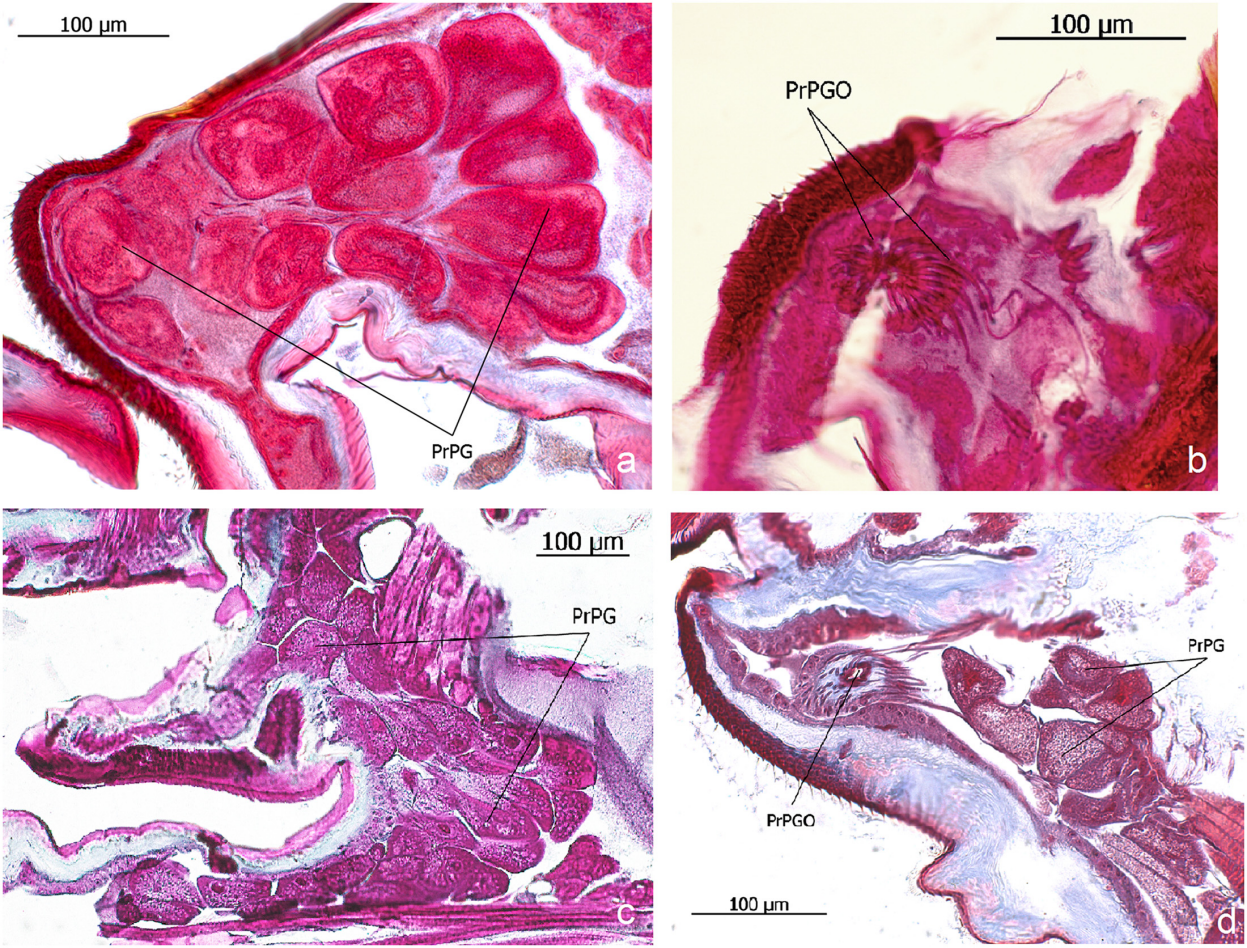
Propharyngeal glands of *Eciton* queens. **a** and **b**: *E*. *hamatum*; **c** and **d**: *E*. *rapax*. Propharyngeal gland (PrPG), opening of the propharyngeal duct cells (PrPGO).

The postpharyngeal gland and the mandibular gland are hypertrophied. The postpharyngeal gland, with its dense body of secretory glandular tubes, fills almost the entire head case (Fig 11). Embedded in this mass of secretory gland tubes (Fig 12) are the two reservoirs of the huge mandibular gland. The reservoir of each mandibular gland stretches from its opening at the base of the mandible all the way to the posterior end of the head-case. The membrane of the gland reservoir is lined with large glandular cells each opening with a short duct into the lumen of the reservoir (Figs 13 and 14). Whelden [13] describes the mandibular gland, but not in its entire extension which can have a length of more than 2000μm. In comparison, the mandibular gland of workers is anatomically similar to that of the queens, but much smaller, not only in absolute size (350–400μm), but also with respect to body proportion (Fig 15). Also, the queen’s maxillary gland is unusually large with more than 150 glandular cells and duct cells on each side. This gland extends from close to the mandible base to the base of the maxilla, and most duct cells open through an invagination of the membrane that appears to constitute a reservoir, but other duct cells open singly through the membrane (Fig 16). We assume this is the gland which Whelden [13] termed “intermediate gland”, but in fact it is the maxillary gland, which is hypertrophied in the *Eciton* queens. We also think that what Whelden called the maxillary gland is in fact the paired propharyngeal gland opening into the mouth cavity in front of the anterior end of the pharynx (Fig 17).

The comparison of all these exocrine glands found in queens with those of *Eciton* workers underlines the enormous special glandular endowment of the queens. Workers do not have the cuticle pores through which the duct cell of numerous glandular cells open and that cover the entire body of the queen. Neither do workers have the many intersegmental glands (except the pygidial and post pygidial glands) [13, 18]. Although Whelden [13] seems to have detected small intersegmental glands in the thorax between thorax and coxae, and in petiole and post petiole, we were unable to find such glands in workers and soldiers. The head glands in workers (postpharyngeal gland, mandibular gland, and propharyngeal gland) are of normal size and the maxillary gland is in fact quite small with approximately 10 gland cells on each side. No glandular cells between antenna and head case and mandibles and head case could be detected. The metapleural gland of queens is considerably larger than that of workers, including soldier caste, but this size difference is still within the body size scale.

We also investigated queens and workers of the ecitonine *Neivamyrmex nigrescens* and *N*. *carolinensis* and found a similar generous endowment of exocrine glands in the queens but not in the workers. Unfortunately the staining of the relatively old histological preparations did not enable us to take reproducible pictures, but they were good enough for us to identify numerous exocrine glands in the dichthadiigynes which are absent in the workers. These observations were supported by SEM micrographs which resemble those taken from *Eciton* queens.

#### Exocrine glands in *Aenictus* queens

**Fig 18:**
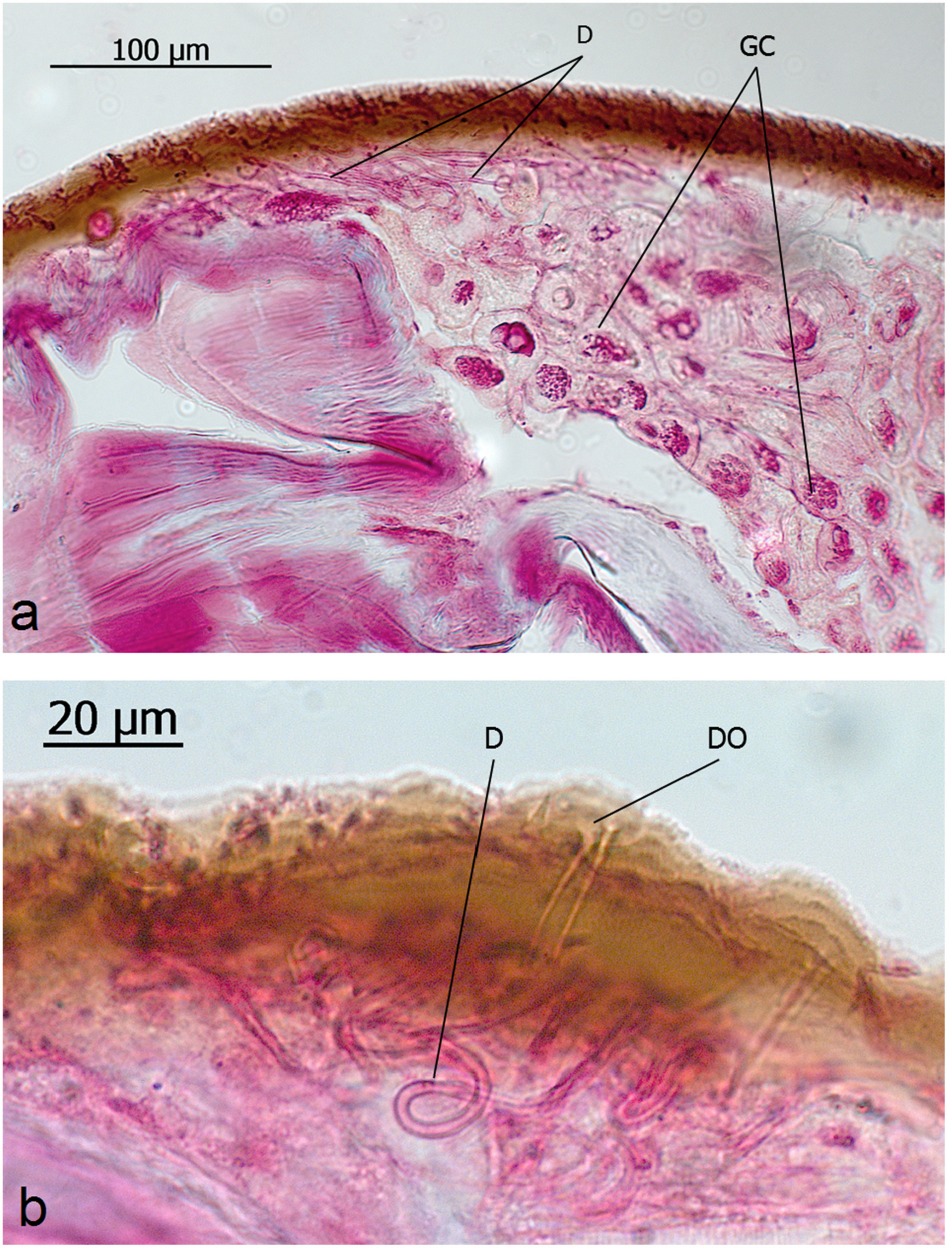
Intersegmental and cuticle glands in queens of *Aenictus* sp. **a** and **b**: Glandular cells (GC), duct cells (D), duct cell opening (DO).

We were able to study only one queen and four workers of an unidentified *Aenictus* species. Although the histological staining was not satisfactory we could identify in the queen intersegmental and cuticle glands which are absent in the workers (Fig 18).

### Intersegmental Glands in Queens of the Legionary Ants *Onychomyrmex* and *Leptanilla*

**Fig 19:**
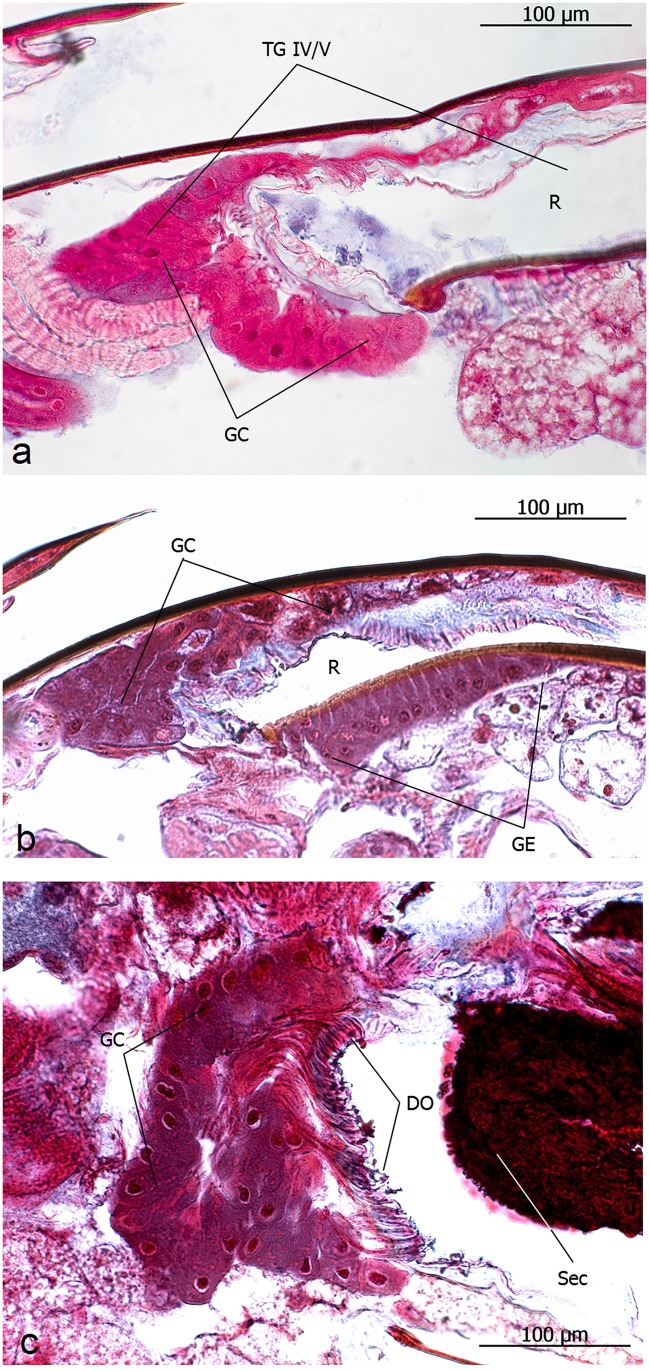
Longitudinal sections through the gaster of queens of *Onychomyrmex hedleyi*. **a**: Intersegmental tergal gland between IVth and Vth tergite (TG IV/V), reservoir (R), glandular cells (GC). **b**: Intersegmental tergal gland between VIth and VIIth tergite (pygidial gland) with glandular epithelium in the VIIth tergite (GE). **c**: Large sternal gland between VIIIth and IXth internal sternites. Duct cell openings (DO), secretion (Sec).

**Fig 20:**
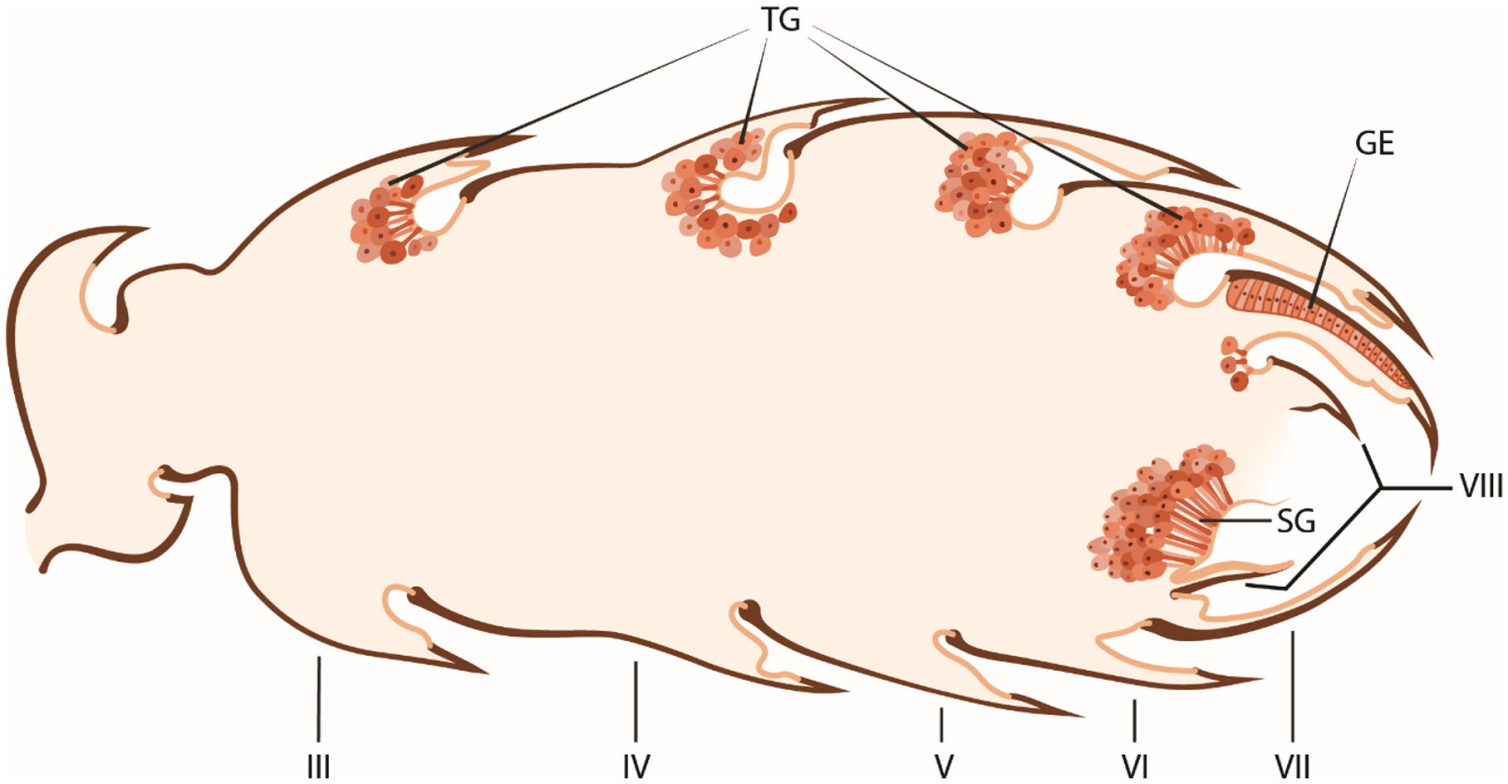
Schematic drawing showing an overview of the intersegmental tergal and sternal glands in the gaster of *Onychomyrmex* queens. Tergal glands (TG), glandular epithelium (GE), sternal gland (SG). Roman numerals indicate abdominal segments.

The ant genus *Onychomyrmex* belongs to the primitive ponerine tribe Amblyoponini. Its species are known to have an army ant or legionary life style ([19]; Robert Taylor personal communication; Hölldobler unpublished observations), in which both mass foraging for small arthropods and colony emigration are highly coordinated. The colonies are monogynous and the queens are dichthadiiform. The workers’ trail pheromone gland is a single cluster of glandular cells, located at the median line between the fifth and sixth abdominal sternites [20]. This gland is absent in the queen. However, the queen is richly endowed with intersegmental glands, consisting of paired clusters of glandular cells with duct cells penetrating the intersegmental membranes between tergites III and IV, IV and V, V and VI, and VI and VII (Fig 19a). In addition there is a large glandular epithelium in tergite VII (Fig 19b) and a few glandular cells between tergites VII and VIII. Furthermore we found a large sternal gland between segment VIII and IX (Fig 19c; for overview see Fig 20). None of these glands are found in the workers, except the gland between tergites VI and VII (pygidial gland) which is very small in the workers with no glandular epithelium in tergite VII. We found these queen glands in four *Onychomyrmex* species, *O*. *hedleyi*, *O*. *doddi*, and two not yet unequivocally described species (Robert Taylor, personal communication).

**Fig 21:**
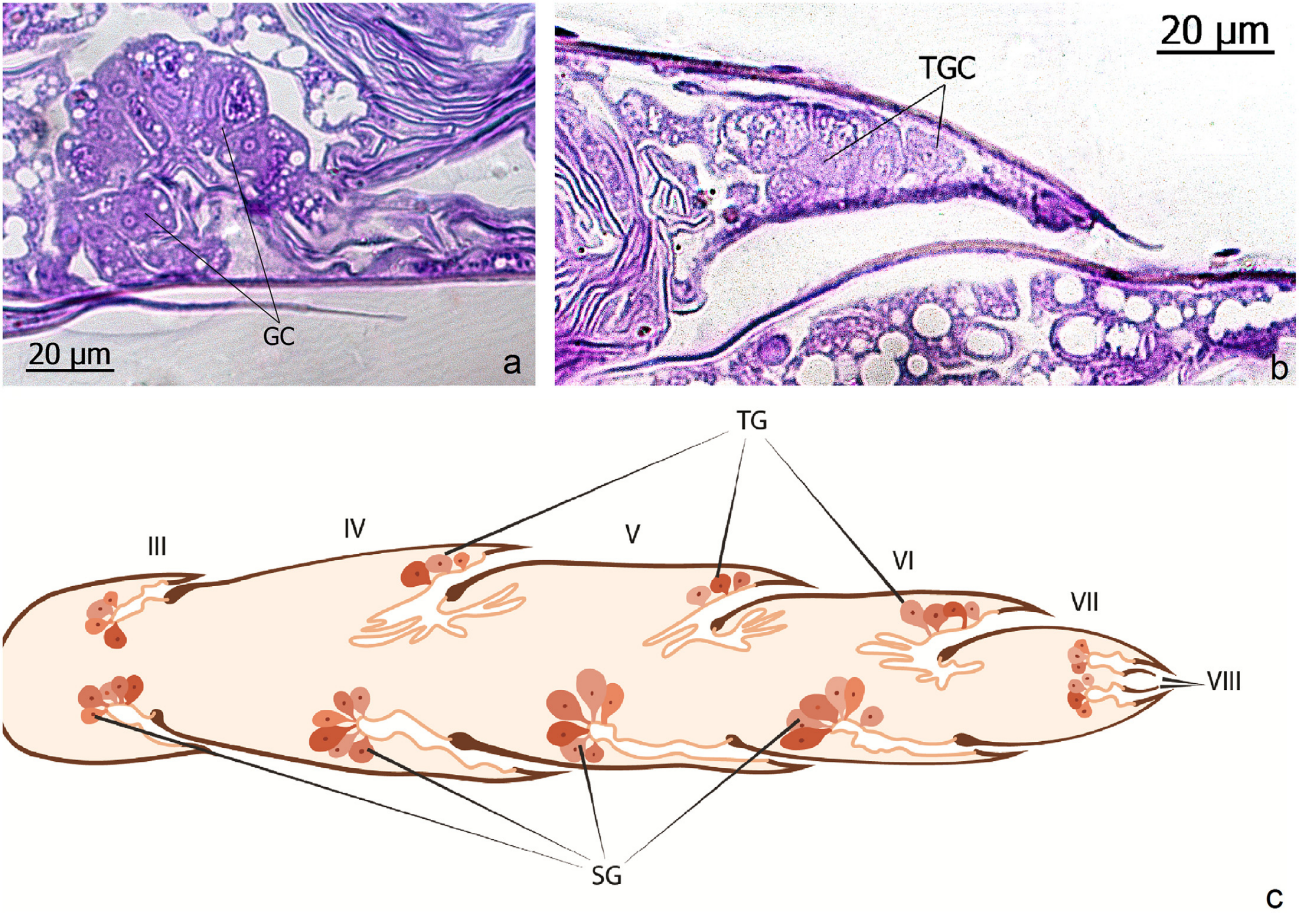
Longitudinal sections through the gaster of queens of *Leptanilla japonica*. **a**: Intersegmental sternal gland cell (GC). **b**: Intersegmental tergal gland cells (TGC). **c**: Schematic illustration showing an overview of the intersegmental tergal and sternal glands in the gaster of *Leptanilla* queens. Tergal glands (TG), sternal glands (SG). Roman numerals indicate abdominal segments.

The genus *Leptanilla* belongs to the small subfamily Leptanillinae. Based on the external morphology of the adults, particularily the dichthadiiform queens, it has been suggested that the leptanilline ants have a legionary mode of life [21]. Masuko [12] was able to collect colonies of *Leptanilla japonica* in Japan, and his observations confirmed an army ant like natural history of this species. The workers prey on centipedes and follow trails when foraging and during colony migrations. Hölldobler et al. [22] identified a special sternal gland in *Leptanilla* workers which most likely serves as trail pheromone gland. This gland is absent in the queen. As already reported in detail [22], the queen is endowed with a remarkable battery of abdominal exocrine glands that are absent in the workers. In the current study we complement these previous findings. Five intersegmental glandular complexes occur pairwise pleurotergally and pleurosternally between abdominal segments III, IV, V, VI and VII (Fig 21a and 21b). The glandular clusters of tergal and sternal glands consist of large cells which are drained by short ducts that penetrate the intersegmental membranes. Fig 21c shows an overview of these abdominal exocrine glands of *Leptanilla* queens.

## Discussion

This study reexamines and extends the investigation of the exocrine glands of queens in army ants and other legionary ant species which are monogynous with dichthadiiform queens. Such highly modified queens are characterized by the possession of a wingless alitrunk, a huge gaster, and an expanded post petiole [23]. Such an exuberance of exocrine glands present in dichthadiigynes has not been found in queens of any other ant species. We predict future investigations will also reveal a rich exocrine gland endowment in the dichthadiiform queens of the other army ant genera *Labidus*, *Nomamyrmex*, *Cheliomyrmex* and *Dorylus*, and in some of the other legionary ant genera with dichthadiiform queens such as *Simopelta*.

Interestingly, in a preliminary survey of some legionary ant species which have monogynous colonies with an ergatoid, but not dichthadiiform, queen, we did not find such exuberance of queen specific exocrine glands. We investigated *Cerapachys turneri* (Queensland) and an unidentified *Leptogenys* species from the Shimba Hills in Kenya. Of course colony size varies a great deal in the genus *Leptogenys*. However, even in *Leptogenys* species with large colonies (such as *L*. *distinguenda*, [24]) that exhibit all behavioral traits of “true” army ants, except that their queens are not dichthadiiform, the queens do not possess more distinct and better developed exocrine glands than workers do (U. Maschwitz personal communication). They may have a better developed glandular epithelial lining than the workers do. This is the case in the African Matabele ants (*Megaponera analis*, formerly called *M*. *foetens)*, also a legionary ant species with monogynous colonies that have an ergatoid, but not dichthadiiform queen, and a relatively large worker force for a ponerine species, ranging from 500 to about 1500 workers (for review see [25]). In *M*. *analis* workers and queens do not differ in their glandular morphology, except for a striking difference in the epidermal epithelium. In the workers the epidermis is a thin layer of collapsed cells, whereas in the queen it is a well-developed glandular epithelium with large nuclei and many vacuoles. The cuticle of the queen is penetrated by a dense network of dermal gland ducts. It has been proposed that this glandular epithelium, lining the entire body, may be the source of a queen signal that makes the queen highly attractive to workers [26]. Indeed, the *M*. *analis* queen is often surrounded by a large retinue of workers and behavioral experiments demonstrated that her attractiveness is based on chemical signals. Although it is rare in colonies of ponerine species, the occurrence of worker retinue around the queen has been described in a number of ant species, e.g. in the weaver ants (*Oecophylla)* [15], the leaf cutter ants of the genus *Atta* [16], the fire ants *Solenopsis invicta* [27] and several other phylogenetically advanced species that form large monogynous colonies [28]. In none of these species, however, are the queens endowed with such an exuberance of exocrine glands, as described here for dichthadiiform queens. Without doubt army ant queens are highly attractive to workers; the worker retinues around queens are impressive [10]. But this cannot be the sole explanation for such a luxurious endowment of queens with exocrine glands.

We think the answer lies in the very specific mode of reproduction in army ants and possibly also in other legionary ants with dichthadiiform queens.

Army ants reproduce by fission. Reproduction in such colonies involves the production of relatively few daughter queens and a large number of males. The new queens are inseminated by males from other colonies, and one of the mated queens leaves the parental colony with a large group of workers. The other moiety of workers stays with the old queen, or if her fertility wanes, the workers may accept one of the other young queens. The remaining young queens will be expelled from the colony and will perish. Franks and Hölldobler [14] hypothesized that during this colony fission workers should select the potentially most vigorous and productive queens, and most likely the workers choose the queen on the basis of chemical queen signals. Extreme competition among the young inseminated queens should be expected and such competition obviously favors the selection of individuals which are better endowed with exocrine glands. Consequently, such selection may have led to a run-away evolution of exocrine glands in army ant queens.

Interestingly, also the males of ecitonine army ants are luxuriously endowed with exocrine glands [29]. When they enter the foreign nest they often shed their wings (Carl Rettenmeyer, personal communication) and are followed by a retinue of workers. Franks and Hölldobler [14] suggested that the males have, like the reproductive females, evolved under the influence of sexual selection the same channels of communication to advertise their potential fitness to the workers. After all, the workers not only control which of the females will become the new queen, but also which males will have access to the virgin females.

Unfortunately nothing is specifically known about colony reproduction in *Onychomyrmex* and *Leptanilla*, but we assume that also in these genera colonies reproduce by colony fission after alate males have entered foreign conspecific colonies and mated with young queens. Perhaps the males will also be endowed with many exocrine glands not found in males of ant species whose queens are not dichthadiiform. It is reasonable to assume that also in colonies of *Onychomyrmex* and *Leptanilla* species the workers are the selective agents that choose the future queens and their mating partners based on an assessment of potential fitness. The outfit with exocrine glands might be a fitness indicator.

As far as we know dichthadiiform queens never leave the nest for mating. This is quite different from many other ant species which have wingless, but not dichthadiiform female reproductives. In such species, for example of the genera *Diacamma* or *Rhitidoponera v*irgin females exhibit “sexual calling” behavior outside the nest during which they release a sex pheromone and attract winged males for mating [30, 31, 32]. In a few species the males enter foreign conspecific colonies and mate with young wingless females inside the nest, but there is no control by workers; these colonies have many reproductive females [33].

It has been suggested that the army ant queens’ rich endowment with exocrine glands has evolved because these colonies frequently migrate, an event during which the colony risks losing the queen. The queen is the most precious member of the colony and her loss would be fatal to the colony. Therefore the queen’s presence is being powerfully advertised by chemical signals [2, 10, 23]. However, the example of *Megaponera analis* has demonstrated that signaling the queen’s presence and attractiveness does not require such an exuberant exocrine gland system as found in army ants and other ant species with dichthadiiform queens. In this context it would be very interesting to investigate the glandular system of queens in some of the subterranean army ant species which lead a hypogaeic life. Using palm oil baits Berghoff et al [34, 35] monitored the hypogaeic movements of *Dorylus (Dichthadia) laevigata* in Sabah (Malaysia, Borneo). Unlike epigaeic army ants which only very rarely reuse old foraging trails, *D*. *laevigata*, *colonies* employ a quite stable hypogaeic trunk trail system. The same foraging system is used for extended periods of time, which implies these *D*. *laevigata* colonies are quite stationary, also confirmed by genetic data [35], and a stationary phase does not regularly alternate with a nomadic phase as is typically the case in epigaeic army ants. We predict that also in these army ant species that do not exhibit regular nomadism, the dichthadiiform queens will be equally luxuriously endowed with exocrine glands.

Maybe we have to reconsider the definition of the term “army ant syndrome” and rather speak of a “dichthadiigyne syndrome”. The “dichthadiigyne syndrome” comprises a special mating system exhibited in the majority of genera of doryline ants and some other ant genera. And a major feature of this syndrome is an exuberance of exocrine glands specific to queens which most likely evolved by a special kind of sexual selection mediated by workers.

Hopefully this paper will stimulate future studies which will support or reject this hypothesis.

## References

[1] Schneirla TC. Army ants: A study in social organization. San Francisco: WH Freeman and Company; 1971.

[2] Gotwald WH Jr. Army ants: The biology of social predation. Ithaca and London: Comstock Publishing Associates, Cornell University Press; 1995.

[3] Brady SG. Evolution of the army ant syndrome: The origin and long-term evolutionary stasis of a complex of behavioral and reproductive adaptations. Proc. Natl. Acad. Sci. U.S.A. 2003; 100: 6575–6579. 12750466 10.1073/pnas.1137809100 PMC164488

[4] Kronauer DJC, Berghoff SM, Powell S, Denny AJ, Edwards KJ, Franks NR, et al A reassessment of the mating system characteristics of the army ant *Eciton burchelli*. Naturwissenschaften 2006; 93: 402–406. 16676159 10.1007/s00114-006-0121-2

[5] Bolton B. Army ants reassessed: The phylogeny and classification of the doryline section (Hymenoptera: Formicidae). J. Nat. Hist. 1990; 24: 1339–1364.

[6] Brady SG, Ward PS. Morphological phylogeny of army ants and other dorylomorphs (Hymenoptera: Formicidae). Syst. Entomol. 2005; 30: 593–618.

[7] Bolton B. Synopsis and classification of Formicidae. Mem. Am. Entomol. Inst. 2003; 71: 1–370.

[8] Brady SG, Fisher BL, Schultz TR, Ward PS. The rise of army ants and their relatives: Diversification of specialized predatory doryline ants. BMC Evol. Biol. 2014; 14: 93 doi: 10.1186/1471-2148-14-93 24886136 10.1186/1471-2148-14-93 PMC4021219

[9] Rettenmeyer CW. Behavioral studies of army ants. Sci. Pap. Univ. Kansas Nat. Hist. Mus. 1963; 44: 281–465.

[10] Rettenmeyer CW, Topoff H, Mirenda J. Queen retinues of army ants. Ann. Entomol. Soc. Am. 1978; 71: 519–528.

[11] Schneirla TC, Reyes AY. Emigration and related behavior in two surface-adapted species of the old world doryline ant *Aenictus*. Anim. Behav. 1969; 17: 87–103. 10.1016/s0003-3472(66)80022-2 5918237

[12] Masuko K, Behavior and ecology of the enigmatic ant *Leptanilla japonica* Baroni Urbani (Hymenoptera: Formicidae: Leptaniillinae). Insectes Soc. 1990; 37: 31–57.

[13] Whelden RM. The anatomy of the adult queen and workers of army ants *Eciton burchelli* Westwood and *E*. *hamatum* Fabricus (Hymenoptera: Formicidae). J. N.Y. Entomol. Soc. 1963; 71: 14–30, 90–115, 158–178, 246–261.

[14] Franks NR, Hölldobler B. Sexual competition during colony reproduction in army ants. Biol. J. Linn. Soc. 1987; 30: 229–243.

[15] Hölldobler B, Wilson EO. Queen control in colonies of weaver ants (Hymenoptera: Formicidae). Ann. Entomol. Soc. Am. 1983; 76: 235–238.

[16] Hölldobler B, Wilson EO. The leafcutter ants: Civilization by instinct. New York: WW Norton and Company; 2011.

[17] Rathmayer W. Methylmethacrylat als Einbettungsmedium für Insekten. Experientia 1962; 18: 47–48. 14490529 10.1007/BF02136658

[18] Hölldobler B, Engel H. Tergal and sternal glands in ants. Psyche 1978; 85: 285–330.

[19] Brown WL. Contributions toward a reclassification of the Formicidae. III. Tribe Amblyoponini (Hymenoptera). Bull. Mus. Comp. Zool. 1960; 122: 145–230.

[20] Hölldobler B, Engel H, Taylor RW. A new sternal gland in ants and its function in chemical communication. Naturwissenschaften 1982; 69: 90–91.

[21] Wilson EO. The insect societies. Cambridge: Harvard University Press; 1971.

[22] Hölldobler B, Palmer JM, Masuko K, Brown WL. New exocrine glands in the legionary ants of the genus *Leptanilla* (Hymenoptera: Formicidae: Leptanillinae). Zoomorphology 1989; 108: 255–262.

[23] Hölldobler B, Wilson EO. The ants. Cambridge: Harvard University Press; 1990.

[24] Witte V, Maschwitz U. Raiding and emigration dynamics in the ponerine army ant *Leptogenys distinguenda* (Hymenoptera, Formicidae). Insectes Soc. 2000; 47: 76–83.

[25] Peeters C. Monogyny and polygyny in ponerine ants with and without queens In: Keller L, editor. Queen number and sociality in insects. Oxford: Oxford University Press; 1991 pp. 235–261.

[26] Hölldobler B, Peeters C, Obermayer M. Exocrine glands and attractiveness of the ergatoid queen in the ponerine ant *Megaponera foetens*. Insectes Soc. 1994; 41: 63–72.

[27] Fletcher DJC, Ross KG. Regulation of reproduction in eusocial Hymenoptera. Annu. Rev. Entomol. 1985; 30: 319–343.

[28] Passera L. L’organisation sociale des fourmis. Toulouse: Privat; 1984.

[29] Hölldobler B, Engel-Siegel H. Tergal and sternal glands in male ants. Psyche 1982; 89: 113–132.

[30] Hölldobler B, Bartz B. Sociobiology of reproduction in ants In: Hölldobler B, Lindauer M, editors. Experimental behavioral ecology and sociobiology. Stuttgart: Gustav Fischer Verlag; 1985 pp. 237–257.

[31] Nakata K, Tsuji K, Hölldobler B, Taki A. Sexual calling by workers using the metatibial gland in the ant, Diacamma sp., from Japan (Hymenoptera: Formicidae). Journal of Insect Behavior 1998; 6: 869–877.

[32] Hölldobler B, Wilson EO. The superorganism. New York, London, W. W. Norton & Company; 2009.

[33] Peeters C, Crewe R. Worker reproduction in the ponerine ants *Ophthalmopone berthoudi*: An alternative form of eusocial organization. Behav. Ecol. Sociobiol. 1985; 18: 29–37.

[34] Berghoff SM, Weissflog A, Linsenmair KE, Hashim R, Maschwitz U. Foraging of a hypogaeic army ant: A long neglected majority. Insectes Soc. 2002; 49: 133–141.

[35] Berghoff SM, Gadau J, Winter T, Linsenmair KE, Maschwitz U. Sociobiology of hypogaeic army ants: Characterization of two sympatric *Dorylus* species on Borneo and their colony conflicts. Insectes Soc. 2003; 50: 139–147.

